# Involvement of Serotonin Transporter Gene Polymorphisms (5-HTT) in Impulsive Behavior in the Japanese Population

**DOI:** 10.1371/journal.pone.0119743

**Published:** 2015-03-16

**Authors:** Michio Nomura, Masayuki Kaneko, Yasunobu Okuma, Jun Nomura, Ichiro Kusumi, Tsukasa Koyama, Yasuyuki Nomura

**Affiliations:** 1 Graduate School of Education, Kyoto University, Yoshida Hon-machi, Sakyo-ku, Kyoto, Japan; 2 Department of Biochemistry, Graduate School of Biomedical and Health Sciences, University of Hiroshima, Minami-ku, Hiroshima, Japan; 3 Department of Pharmacology, Faculty of Pharmaceutical Sciences, Chiba Institute of Science, Choshi, Japan; 4 Department of Biomedical Research, Graduate School of Biomedical and Health Sciences, University of Hiroshima, Minami-ku, Hiroshima, Japan; 5 Department of Psychiatry, Graduate School of Medicine, Hokkaido University, Sapporo, Japan; 6 Department of Pharmacology, Kurume University School of Medicine, Kurume, Fukuoka, Japan; RIKEN Brain Science Institution, JAPAN

## Abstract

The serotonergic pathway has been implicated in the pathogenesis of impulsivity, and sensitivity to aversive outcomes may be linked to serotonin (5-HT) levels. Polymorphisms in the gene that encodes the serotonin transporter (5-HTT), which have differential effects on the level of serotonin transmission, display alternate responses to aversive stimuli. However, recent studies have shown that 5-HT does not affect motor function, which suggests that the functioning of the serotonin-transporter-linked polymorphic region (5-HTTLPR) does not directly affect the behavioral regulatory process itself, but instead exerts an effect via the evaluation of the potential risk associated with particular behavioral outputs. The aim of the present study was to examine the effect of specific 5-HTTLPR genotypes on the motor regulatory process, as observed during a Go/Nogo punishment feedback task. 5-HTT gene-linked promoter polymorphisms were analyzed by polymerase chain reaction, using lymphocytes from 61 healthy Japanese volunteers. Impulsivity was defined as the number of commission errors (responding when one should not) made during a Go/Nogo task. We found that the s/s genotype group made fewer impulsive responses, specifically under aversive conditions for committing such errors, compared to those in the s/l group, without affecting overall motor inhibition. These results suggest that 5-HTTLPRs do not directly affect the behavioral regulatory process itself, but may instead exert an effect on the evaluation of potential risk. The results also indicate that under such aversive conditions, decreased expression of 5-HTT may promote motor inhibitory control.

## Introduction

Impulsivity is characterized by actions based on sudden desires and whims rather than on careful thought. The concept of impulsivity covers a wide range of actions that are poorly conceived, prematurely expressed, unduly risky, inappropriate to the situation, and often result in undesirable outcomes [1]. Impulsivity plays a role in normal behavior, but can also occur in a pathological form; therefore, impulsivity has been viewed as a key feature that can aid in the risk assessment of violent and aggressive behavior [2]. In addition, impulsivity acts as a key trait of certain psychological disorders, including pathological gambling, eating disorders, and many other uncontrollable behaviors [3,4]. Thus, it has been said that impulsivity incorporates failure of response inhibition, novelty seeking, and an inability to delay gratification [5]. Amongst the different forms of impulsivity, motor impulsivity is the inability to inhibit planned or ongoing actions, and is usually associated with quick, possibly aggressive, reactions with little regard for consequences. Furthermore, such rash actions of emotion-based dispositions are characterized by two subtypes; positive urgency, which is the tendency to engage in rash action in response to extreme positive affect, and negative urgency, which is the tendency to engage in rash action in response to extreme negative affect [6,7].

Neurobiological findings suggest that certain brain regions are involved in impulsive behavior, with specific brain regions involved in specific behavioral manifestations; genetics are also believed to act as an important inducing factor. A series of psychopharmacological studies implicated several neurochemical pathways in the pathogenesis underlying impulsivity, including the serotonergic pathway. Indeed, it has been suggested that dysfunction in serotonin (5-hydroxytryptamine, 5-HT) neurotransmission may contribute to behavioral disorders that are characterized by motor impulsivity in humans, nonhuman primates, and rodents [8,9]. The relationship between sensitivity to aversive outcomes and 5-HT levels is a consistent biological finding [10]. Depletion of 5-HT reportedly enhances behavioral and brain responses to punishment and other aversive stimuli, with findings suggesting that 5-HT can modulate the impact of punishment-related signals on learning and emotion [11], and can lead to a selective reduction in punishment-induced inhibition without affecting overall motor response inhibition [12].

The serotonin transporter (5-HTT) is one of the major modulators of serotonergic neurotransmission because it is responsible for the reuptake of 5-HT at nerve terminals, and thus determines the magnitude and duration of 5-HT signaling. The 5-HTT gene-linked polymorphic region, a portion of the *SLC6A4*, has been shown to differentially modulate the transcription of *SLC6A4*. This polymorphic region is known as the serotonin-transporter-linked polymorphic region (5-HTTLPR) [13]. Lesch et al. [13] reported that the short allele (S) polymorphic variant of *SLC6A4* decreases the reuptake of 5-HT in the lymphoblasts due to lower expression of 5-HTT mRNA in comparison to the long allele (L) variant. In comparison to L-allele carriers, the biochemical outcome associated with the S allele variant is thought to result in enhanced neural processing of aversive environmental cues, along with increased amygdala activation during the presentation of fearful faces. This in turn could relate to the attentional bias for negative stimuli that is associated with S-allele carriers, along with the increased risk of neuroticism [14,15]. The S allele could also account for mothers’ levels of positive parenting, an effect mediated by children’s self-control [16], and emotional regulation disorders in terms of early and adult life adversities [17], suggesting that emotional events in gene-environment interactions with the 5-HTTLPR could affect impulsive behavior. Furthermore, a meta-analysis showed that S-allele carriers have an increased susceptibility to emotional disorders and increased reactivity of the amygdala when exposed to negative environmental stimuli [18]. For this reason, although the existing data are conflicting and have been met with mounting criticisms and challenges [19,20], most of the research has focused on the negative impact of 5-HTTLPR polymorphisms [see reviews by 21,22].

Interestingly, it appears that motor functioning is not influenced by 5-HTTLPR polymorphisms, as shown by several studies that used the continuous performance test (CPT) and stop-signal task (SST), both of which involve withholding an ongoing or dominant motor response [23,24]. These observations suggest that 5-HTTLPR polymorphisms do not directly affect the behavioral regulatory process itself, but may instead exert an effect via the presence of the potential risk associated with a particular behavioral output. Moreover, as effective motor inhibition is associated with neuroticism, and cautious people become even more cautious after an impulsive response is punished [25,26], it was predicted that s/s carriers might have an increased ability to inhibit their motor responses appropriately in a risky context.

The effect of 5-HTTLPR polymorphisms on motor impulsivity remains poorly understood. Thus, the aim of the present study was to examine the effect of specific 5-HTTLPR genotypes on the motor regulatory process during a Go/Nogo punishment feedback task. Since S-allele carriers are more sensitive to the negative affect elicited by punishment, this may cause them to be less impulsive than L-allele carriers. Therefore, we hypothesized that the motor response to negative stimuli would vary as a function of the 5-HTTLPR gene polymorphism such that S-homozygous individuals would have an enhanced ability to inhibit their responses correctly compared to L carriers.

## Materials and Methods

### Participants

Sixty-one Japanese participants with normal or corrected-to-normal vision were enrolled in this study. Participants were recruited from among the laboratory, office, and hospital staff of Hokkaido University School of Medicine and from the student body of the College of Medical Technology, Hokkaido University. The experimental procedure was approved by an Internal Review Board at Chiba Institute of Science (Human Research Ethics. Committee) and conducted in accordance with the Declaration of Helsinki. All participants underwent an interview to exclude individuals with psychiatric disorders, as classified by the Diagnostic and Statistical Manual of Mental Disorders, Fourth Edition (DSM-IV) [27]. Participants included 34 males and 27 females, with a mean age of 29.0 ± 1.1 years (mean ± standard error). On average, participants had 6.0 ± 0.9 years of university education. All participants signed a statement declaring they were drug-free for at least 4 weeks prior to blood sampling and that they had no history of physical or psychiatric illness. Each participant gave written informed consent to participate after receiving a detailed description of the study.

### Behavioral procedures: assessment of disinhibition

In order to evaluate motor impulsivity in an aversive context, we adopted the reinforcement punishment-reward task, Go/Nogo [8,28]. This task uses punishments or rewards to promote response activations or suppressions [29]. Participants were required to press the computer key when a “Go” stimulus appeared on the screen (randomly assigned numbers) and to withhold this response when a “Nogo” stimulus appeared on the screen (other randomly assigned numbers). Only one type of stimulus was presented at a time, and the participants learned the process through trial and error.

**Fig 1:**
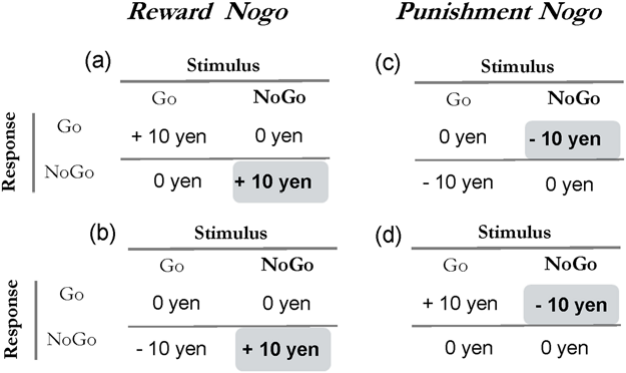
Response-outcome contingencies in the experimental conditions. In the “reward Nogo” conditions, participants were rewarded when they withheld responses to the “Nogo” stimuli: (*a*) under the reward-reward condition, participants were also rewarded for responding to the “Go” stimuli, while (*b*) under the punishment-reward condition, participants were punished for withholding responses to the “Go” stimuli. In the “punishment Nogo” conditions, participants were punished when they responded to the “Nogo” stimuli: (*c*) under the punishment-punishment condition, participants were also punished for withholding responses to the “Go” stimuli, while (*d*) under the reward-punishment condition, participants were rewarded for responding to the “Go” stimuli.

The Go/Nogo task was assessed using four different conditions, and the order of conditions differed across participants. All conditions contained four different sets of stimuli that were grouped into four different blocks with one set for each condition. The frequency of the “Go” and “Nogo” stimuli was set at a ratio of 1:1. Under the reward-reward (RR) condition, participants were rewarded for both responding to the “Go” stimuli and withholding responses to the “Nogo” stimuli. Under the punishment-reward (PR) condition, participants were punished for withholding responses to the “Go” stimuli, while being rewarded for withholding responses to the “Nogo” stimuli. Both of these conditions were considered “reward Nogo” conditions since participants were rewarded when they withhold responses to a “Nogo” stimulus. Under the punishment-punishment (PP) condition, participants were punished for both withholding responses to the “Go” stimuli and responding to the “Nogo” stimuli. Under the reward-punishment (RP) condition, participants were rewarded for responding to the “Go” stimuli, but punished for responding to the “Nogo” stimuli. Both of these conditions were considered “punishment Nogo” conditions since participants were punished when they pushed the button in response to a “Nogo” stimulus (Fig. 1). In this study, we focused on these conditions, as motor impulsivity is defined as responding to “Nogo stimuli” [30], otherwise looking at alternative classification of “Go” stimuli.

Practice trials were carried out in both the first session (12 trials) and second session (15 trials), with “Go” and “Nogo” stimuli provided in the ratio of 2:1, respectively. The trial task was performed in the presence of the experimenter to establish the dominant response set for rewards [31]. After the practice trials, the experimenter left the room to allow the participant to concentrate on the task. Participants were randomly assigned one of 24 possible presentation orders of the four conditions. The conditions consisted of two sessions, in accordance with the task procedure previously described [28]: in the first session, 12 numbers (6 “Go,” 6 “Nogo”) were repeated five times in a randomized order for a total of 60 trials. In the second session, 14 different numbers (7 “Go,” 7 “Nogo”) were repeated six times in a randomized order for a total of 84 trials. Each stimulus was presented on the screen for 800 ms, with a 1300-ms inter-trial interval. Reward was classified as a correct response and denoted by the word “CORRECT” appearing on the computer screen for 1000 ms, as well as by participants receiving 10 yen as monetary feedback. Punishment was classified as a wrong response and denoted by the word “WRONG” appearing on the computer screen for 1000 ms, as well as by subtracting 10 yen from participants’ earnings. At the end of each condition, the experimenter reentered the room to explain the next condition.

Dependent measures for this task included both omission errors (not responding to the “Go” stimuli) and commission errors (not inhibiting responses to the “Nogo” stimuli); a larger number of commission errors indicated greater difficulty in inhibiting impulsive behavior. Commission and omission errors were calculated separately across the 144 trials under each condition.

### Genotyping

Genomic DNA was extracted from lymphocytes isolated from whole blood samples (20 ml) by standard methods. Briefly, 3.8% Na-citrated whole blood samples were centrifuged at 1,000x g for 20 min and the middle layer was separated for lymphocyte preparation by further centrifugation. For genotyping of 5-HTTLPR polymorphisms, polymerase chain reactions (PCRs) were performed using the primers, 5'-GGCGTTGCCGCTCTGAATGC-3' and 5'-GAGGGACTGAGCTGGACAACCAC-3', in a solution containing 20 ng genomic DNA, 1 mM MgSO_4_, 0.3 mM deoxynucleotide triphosphates, 0.3 M of each primer, Pfx Amplification Buffer, and 0.5 U of Platinum Pfx DNA Polymerase (Invitrogen Corp., Carlsbad, CA). The protocol used was as follows: initial denaturation at 94°C for 2 min; 35 cycles of denaturation at 94°C for 30 s, annealing at 61°C for 30 s, and extension at 68°C for 1 min; and final extension for 5 min at 68°C. The PCR products were then analyzed in a 2% agarose gel stained with ethidium bromide. The amplification product was 528 bp for the L allele and 484 bp for the S allele [13]. Individuals carrying double copies of the S allele (s/s genotype), the S and L alleles (s/l genotype), and double copies of the L allele (l/l genotype) were identified; however, only responses across the s/s (34) and s/l (26) genotypes were compared in order to match population prevalence, as the proportion of l/l carriers in the Asian population is small [32,33].

### Statistical Analysis

For the Go/Nogo task, square root transformations were applied to the mean percentages of commission and omission errors, and these were calculated for each stimulus condition to normalize the positively skewed distributions. Two-way repeated-measures analyses of variance (ANOVAs) were then performed using the subject groups, s/s and s/l, and the task conditions, RR, PP, RP, and PR. Furthermore, to test the hypothesis that response inhibition to negative stimuli would vary as a function of the 5-HTTLPR gene polymorphism, two-way ANOVAs were also performed using the subject groups, s/s and s/l, and the “Nogo” conditions, “punishment Nogo” and “reward Nogo.”

Potential confounding variables such as age, years of education, and gender were assessed separately by two-way analyses of covariance (ANCOVAs) (SPSS 16.0). A statistical significance threshold of *p* < 0.05 was used; confounding variables exceeding this threshold were included as covariates in further statistical analyses.

## Results

### Demographic data and 5-HTT gene polymorphisms

Of the 61 participants, 26 (42.6%) had the s/l genotype and 34 (55.7%) had the s/s genotype, while only 1 (1.6%) had the l/l genotype. The genotype frequencies were in Hardy-Weinberg equilibrium (χ^2^ = 2.57, df = 1, *p* = 0.11). Table 1 presents the demographic data for the s/l and s/s participants. Because there was only 1 participant with the l/l genotype, data from this participant was excluded from further analyses.

**Table 1:** Characteristics of Male and Female subjects sorted by 5-HTTLPR genotypes.

			Mean±S.D.	
			SL	SS
		n	26	34
		Men	0.50	0.59
**Age (years)**			32.4±12.1	25.8± 9.3
**Education (years)**			19.3± 4.0	16.9± 3.8
**Reaction time (ms)**				
	CER	RP	545.9±82.4	543.2±73.9
		PP	535.1±77.8	542.6±72.3
		RR	540.9±76.8	542.1±79.6
		PR	548.3±70.1	549.9±73.3
	HIT	RP	569.7±77.8	546.6±67.4
		PP	545.2±90.7	543.4±76.9
		RR	550.2±92.4	560.7±72.7
		PR	564.2±81.3	551.9±78.2
**Error rate (%)**				
	CER	RP	24.7±13.9	15.9±12.6
		PP	26.4±14.5	18.0±12.6
		RR	26.7±18.1	27.1±13.1
		PR	30.5±14.6	34.9±13.8
	OER	RP	30.8±10.8	30.9±11.4
		PP	26.8±12.2	24.8± 7.7
		RR	21.0±11.4	24.0±11.7
		PR	23.0±13.8	18.7±10.7

HIT, responding to Go stimuli. CER: Commission error rate, responding to NoGo stimuli. OER: Omission error rate, not responding to Go stimuli

### Impulsivity

#### Go/Nogo commission errors

**Fig 2:**
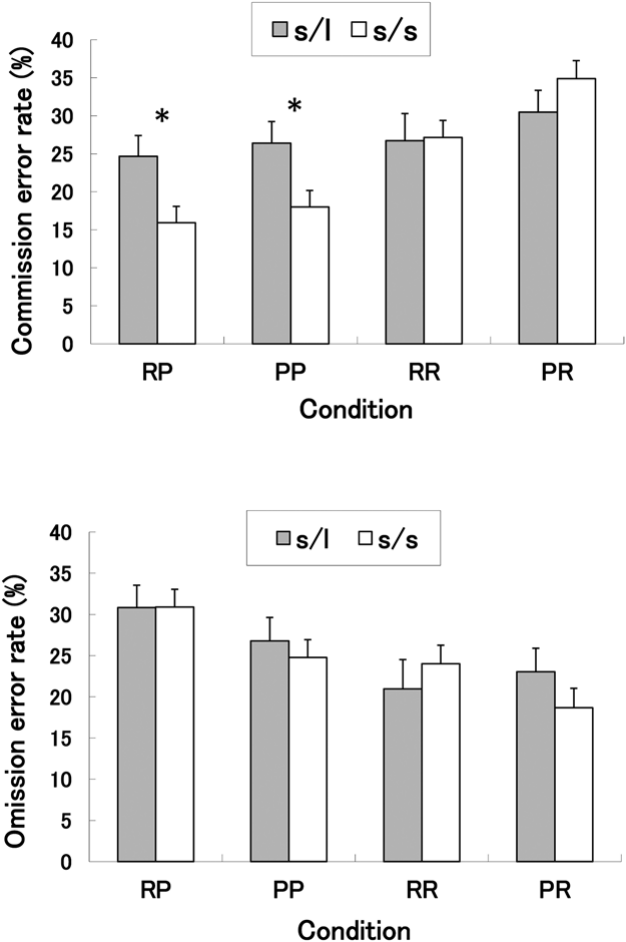
Commission and omission error rates in all conditions. Mean (± standard error) commission error rates (%) (top) and omission error rates (bottom) under four conditions for each of the two allele groups (s/s and s/l). RR: reward-reward condition; PP: punishment-punishment condition; RP: reward-punishment condition; PR: punishment-reward condition. *p < 0.05

The two-way ANOVA of the commission errors between groups revealed a main effect of condition (*F*_3,56_ = 13.56, *p* < 0.001, η_p_^2^ = 0.19) and a genotype × condition interaction (*F*_3,56_ = 4.75, *p* < 0.005, η_p_^2^ = 0.08). As reflected by these interactions, the effect of genotype varied according to condition. Post hoc tests revealed that the s/s group made significantly fewer commission errors compared to the s/l group for both the RP and PP conditions, but not for the RR and PR conditions (Tukey HSD post hoc test, *p* < 0.05) (see Fig. 2). These results suggest that the 5-HTT genotype is associated with a measurement of motor impulsivity in “punishment-Nogo” conditions irrespective of “Go” responses.

**Fig 3:**
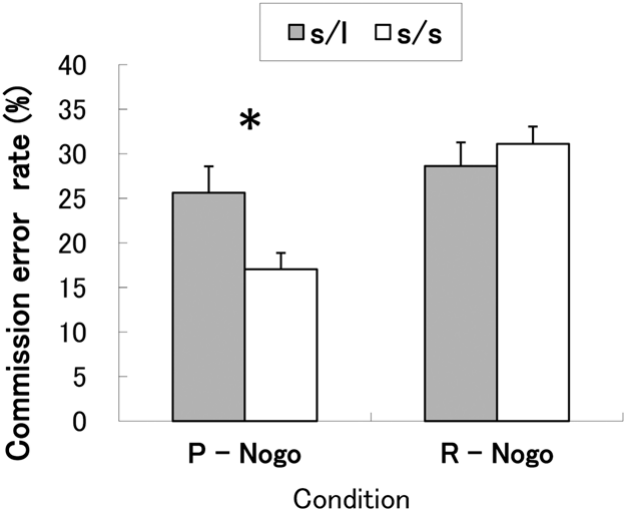
Commission error rates in the “Nogo” conditions. Mean (± standard error) commission error rates (%) under two “Nogo” conditions for each of the two allele groups (s/s and s/l). P-Nogo: “punishment Nogo” conditions, where participants were punished for pushing the button in response to a “Nogo” stimulus; R-Nogo: “reward Nogo” conditions, where participants were rewarded for withholding responses to a “Nogo” stimulus. *p < 0.05.

To test the hypothesis that the motor impulsivity to negative stimuli would vary as a function of the 5-HTTLPR gene polymorphism, a two-way ANOVA of the commission errors was performed using the subject groups and the “Nogo” conditions (“punishment Nogo” vs. “reward Nogo”), which revealed a main effect of genotype (*F*_1,58_ = 26.53, *p* < 0.001, η_p_^2^ = 0.31) and a group × condition interaction (*F*_1,58_ = 11.15, *p* < 0.005, η_p_^2^ = 0.16) (see Fig. 3). This indicates that the s/s group made significantly fewer commission errors compared to the s/l group and suggests that the s/s group had an increased ability to inhibit their responses correctly during the aversive conditions compared to the reward conditions.

#### Go/Nogo omission errors

As shown in Fig. 2, the two-way ANOVA of the omission errors between groups revealed a main effect of condition (*F*_3,56_ = 11.80, *p* < 0.001, η_p_^2^ = 0.17), but did not show a main effect of genotype (*F*_3,56_ = 0.18, *p* > 0.1, η_p_^2^ = 0.03) or a genotype × condition interaction (*F*_3,56_ = 1.50, *p* > 0.1, η_p_^2^ = 0.25), indicating that the 5-HTTLPR had no effect on omission errors. Post hoc tests revealed that the omission errors were higher in the RP condition compared to the PP, RR, and PR conditions for each genotype (Tukey HSD post hoc test, *p* < 0.05). Analysis of the omission errors using the subject groups and the “Nogo” conditions (“punishment Nogo” vs. “reward Nogo”) did not reveal a main effect of genotype (*F*_1,58_ = 0.20, *p* > 1.0, η_p_^2^ = 0.00) or a genotype × condition interaction (*F*_1,58_ = 1.50, *p* > 1.0, η_p_^2^ = 0.25), but did show a main effect of condition (*F*_1,58_ = 22.35, *p* < 0.001, η_p_^2^ = 0.28). Omission errors were higher in the “punishment Nogo” condition compared to in the “reward Nogo” condition (Tukey HSD post hoc test, *p* < 0.05).

Thus, 5-HTTLPR only influenced impulsive responses (commission errors, as described above). These findings were robust after controlling for group differences in age, years of education, and gender.

#### Go/Nogo reaction times (commission errors and hits)

The two-way ANOVA of the reaction time for commission errors between groups did not reveal a main effect of genotype (*F*_3,58_ = 0.11, *p* > 1.0, η_p_^2^ = 0.00), condition (*F*_3,58_ = 1.16, *p* > 1.0, η_p_^2^ = 0.20), or a genotype × condition interaction (*F*_3,58_ = = 0.11, *p* > 1.0, η_p_^2^ = 0.00). Next, we conducted a two-way ANOVA of the reaction time for commission errors using the subject groups and “Nogo” conditions (“punishment Nogo” vs. “reward Nogo”). This analysis did not reveal a main effect of genotype (*F*_1,58_ = 0.1, *p* > 1.0, η_p_^2^ = 0.00), condition (*F*_1,58_ = 1.16, *p* < 0.001, η_p_^2^ = 0.20), or a genotype × condition interaction (*F*_1,58_ = 0.27, *p* > 1.0, η_p_^2^ = 0.005).

The two-way ANOVA of the reaction time for hits between groups did not reveal a main effect of genotype (*F*_3,58_ = 0.13, *p* > 1.0, η_p_^2^ = 0.002), condition (*F*_3,58_ = 1.71, *p* > 1.0, η_p_^2^ = 0.03), or a genotype× condition interaction (*F*_3,58_ = 2.01, *p* > 1.0, η_p_^2^ = 0.03). Then, we conducted a two-way ANOVA of the reaction time for commission errors using the subject groups and the “Nogo” conditions (“punishment Nogo” vs. “reward Nogo”), which did not reveal a main effect of genotype (*F*_1,58_ = 0.13, *p* > 1.0, η_p_^2^ = 0.002), condition (*F*_1,58_ = 1.06, *p* > 1.0, η_p_^2^ = 0.020), or a genotype × condition interaction (*F*_1,58_ = 1.17, *p* > 1.0, η_p_^2^ = 0.02). Overall, reaction times for hits and commission errors showed no statistical differences (Table 1). These findings were robust after controlling for group differences in age, years of education, and gender.

## Discussion

The aim of the present study was to examine the effect of specific 5-HTTLPR genotypes on the motor regulatory process, as observed during the Go/Nogo punishment feedback task. We found that different 5-HTTLPR genotypes have a specific influence on motor impulsivity, whereby s/s carriers made fewer commission errors than s/l carriers did, specifically during the aversive conditions. Our findings correspond to findings from previous studies on motor impulsivity, which have reported that cautious people become even more cautious in terms of motor inhibition during a stop-signal task after an impulsive response is punished [25]. Our results also correspond with the reported association between neuroticism and effective motor inhibition in Go/Nogo tasks involving the probability of receiving punishment [26]. Importantly, the effect of genotype was seen in commission errors, but not in omission errors, suggesting that the effect did not only rely on working memory (WM) load. If WM function differed between the genotypes, the genotype effect should also be seen in omission errors, as they also depend on WM similar to commission errors.

None of these studies addressed the mechanism by which this motor impulsivity occurred. Recently, a series of psychopharmacological studies on impulsivity implicated several neurochemical pathways in the underlying pathogenesis, including the serotonergic pathway. 5-HTT is one of the major modulators of serotonergic neurotransmission, in that it determines the magnitude and duration of 5-HT signaling. We know from previous findings that polymorphisms in the 5-HTT gene-linked region lead to differential 5-HT levels and behavioral effects [13]. In comparison to the L allele, the S allele reportedly decreases clomipramine (non-selective serotonin reuptake inhibitor)-induced prolactin release in healthy humans [34], since prolactin secretion may be stimulated by serotonin, suggesting that acute 5-HT reuptake blockades produce a weaker increase in 5-HT neurotransmission in individuals with the S allele. These and other previous studies have generally grouped S carriers together compared to l/l carriers; however, a few studies, including the present study, have investigated the effects of the s/s and s/l genotypes on serotonergic function.

For instance, antidepressant effect of selective serotonin reuptake inhibitors (SSRIs) such as fluvoxamine [35] and paroxetine [36] were reportedly more effective in depressive patients carrying the s/l allele than in patients carrying the s/s allele. Since SSRIs are thought to exert their effects through the binding of 5-HTT and the inhibition of 5-HT reuptake, and given that the antidepressant effect of SSRIs seems to vary according to the 5-HTTLPR genotype, it was proposed that a different level of expression of 5-HTT might exist between s/s carriers and s/l carriers. l allele is lower in Japanese than in Caucasians; therefore, the antidepressant effect of fluvoxamine can be not as good in Japanese as in Caucasians. The authors investigated whether 5-HTTLPR was associated with the. The present study suggests that fluvoxamine is not less effective in depressive patients carrying the s all

Instead, even in healthy controls, it has been demonstrated that the prolactin response to fenfluramine is significant in s/l carriers, but not in s/s carriers, a response that is dependent upon the density of presynaptic 5-HTTs [37]. Although, as in the S allele frequency was significantly higher in the antidepressant response to fluvoxamine than in the nonresponsive ones in Japanese with major depressive disorder [38], it should be noted that whether pharmacological effects of 5-HT reuptake inhibitors are quantitatively different between s/s and s/l are still controversial and the issue which should be clarified by systematically considering the certain factors: drugs utilized, administration (dose and period of treatment), subjects (age, sex, ethnics etc.) and kind of effect analyzed. In any case, as differential effects of the s/s and s/l genotypes have also been implicated in serotonin-related behavioral phenotypes such as parenting [39], the s/s and s/l genotypes might differentially affect behaviors as well as serotonergic function, and the present data lead to the idea that reduced expression of 5-HTT could facilitate the ability to inhibit motor impulsivity in an aversive context.

Since increased 5-HTT expression in mice is associated with decreased 5-HT transmission [40], the functional consequences of the s/l genotype may disrupt the effective regulation of motor impulsivity in an aversive context due to these individuals having lower 5-HT concentrations in the extracellular space compared to s/s carriers. In line with this observation, Crockett et al. [12] clearly demonstrated that decreased levels of 5-HT through acute tryptophan depletion in humans leads to a selective reduction in punishment-induced inhibition without affecting overall motor response inhibition. Together with the findings of Crockett et al. [12], the present findings show that the punishment-induced conditions for “Nogo” responses, couple with irrespective of the conditions for “Go” responses,, s/s genotype group made fewer impulsive responses, but this effect is not seen in the punishment-induced conditions for “Go” responses (i.e., PR condition), suggesting that serotonin may not affect sensitivity to punishment in general. Overall, the present observations suggest that weaker responses to 5-HT may specifically impair behavioral inhibition in an aversive context.

Interestingly, S-allele carriers demonstrate a greater amygdala response than l/l carriers to fearful faces [19,41], and exhibit elevated levels of amygdala blood flow at rest [42,43], which may promote vigilance to threat. As a key neural structure involved in the rapid integration of aversive inputs and in the regulation of motor output processes [44,45], the amygdala serves as a warning sign for potential social threats [21,46,47], and then facilitates the perceiver’s inhibitory behavior in response to threatening facial emotions [48]. Taking these observation into account, the present data may suggest that the potential role of the s/s allele is to effectively inhibit motor responses, not only under the threat of monetary punishment, but also under conditions that involve more general social threats, e.g., fearful, angry faces and the loss of reputation.

Several recent studies have demonstrated that the S-allele genotype in rhesus macaques is associated with improved cognitive functioning in the delayed phase of the pattern recognition memory task, the delayed match-to-sample task, and the Wisconsin Card Sorting Test [for a review see 49]. Homberg and Lesch [49] focused on the “bright side” of the 5-HTTLPR S allele by explaining that the environment shapes the phenotypic outcome of these fundamentally neutral common genetic factors, possibly leading to negative outcomes, but also having the potential to result in positive behavioral manifestations. Critically, the results of the present study suggest that improved cognitive functioning could interact with certain vulnerabilities, such as oversensitivity to punishment, to contribute to the effective behavioral regulation under certain conditions. As such, these factors may counteract or completely offset the negative consequences of anxiety-related traits. In short, risk alleles of gene polymorphisms (such as the 5-HTTLPR S allele) may exert beneficial, as well as maladaptive effects.

It should be noted that our findings pertain to the Japanese population. Analysis of the population genetics has revealed that approximately 80% of the Japanese population carries the S allele polymorphism [32], while only approximately 40% of Europeans carry this allele [50]. Interestingly, studies on population genetics imply that the population frequency of S allele carriers and the population frequency distribution of cultural collectivism are matched: higher population frequencies of S allele carriers are associated with increases in cultural collectivism [51]. This seems to be consistent with our biological data, in that the relatively effective regulation of behavior in response to punishment that was associated with the S allele may play a major role in the maintenance of social order in Japanese society, which is an example of a collectivistic and “tight” society [52,53] where severe sanctions are imposed on those who deviate from social norms. In turn, this might promote collectivistic cultural norms [54–56]. Needless to say, 5-HTTLPR is one of many genes that may affect our relationship with society/culture; thus, it is worth considering the cultural implications of the relationship between the prevalence of the 5-HTTLPR gene and environmental factors.

Interestingly, although there was no main effect of genotype on this error, the omission error rates were higher in reward-punishment condition compared to other conditions for both genotypes. Under the reward-punishment condition, participants receive a reward if they execute the response, that is, they do not lose any money at Go trial whether they respond or not. On the other hand, during Nogo trial, if they make an error in withholding the response, they lose money. Thus it would be better withholding responses irrespective of trials to avoid punishment, which may lead to increasing tendency to make an omission error during Go trial. In contrast, during PR condition, it would be better execute motor responses in each trial to avoid punishment, which manifested as increasing commission errors. In addition, the present results seems to be consistent with the previous study who showing that the mean of omission error in PR condition of the Go/No-go task were the highest, although it was not statistically significant, with healthy controls [28]. The present study focused on impulsivity, however, it is also important to clarify the inability to execute responses in the Japanese participants have a particular difficulty with [57].

This study has a few limitations. First, our findings need to be replicated in other populations using the same variants. Furthermore, an A/G nucleotide substitution in the L allele, known as the tri-allelic effect, L_G_, which is functionally equivalent to the S allele compared to the L_A_ allele [58,59], should be considered in future studies. Second, the present results are consistent with neuroimaging studies performed in Caucasian populations [41], but not in other Asian populations; Lee and Ham [60] showed that s/l carriers demonstrate a greater amygdala response than s/s carriers to angry faces in Koreans. Since over 80% of Koreans are collectivistic, while only around 50% of Japanese are collectivistic, which is much closer to the prevalence of Western societies [51], these cultural differences might account for the differences. In addition to amygdala, there are motor inhibitory related area such as pre-SMA, parietal regions, and prefrontal cortex [61, 62], which might be involved in serotonergic levels and/or signalling the underlying mechanisms of 5-HTTLPR should be also clarified. Lastly, because we focused on this candidate gene, our sample size was not as large as the sample sizes in subjective questionnaire-based studies or genome-wide association studies; however, our sample size was similar to other studies using behavioral measures.

In conclusion, to our knowledge, the present study is the first human genetics study to show that 5-HTTLPR affects motor impulsivity in response to aversive outcomes, thus suggesting that motor inhibitory control is promoted by 5-HT. Considering the present findings were observed in a Japanese population, one important agenda for future work is to test if 5-HTTLPR variability across regions and ethnic groups has a similar effect. Further, it will be important to test the potential effects of both individual and a wider range of genes that pertain not only to the serotonergic system, but also to the other candidate genes, in a large longitudinal study to clarify the complex and bidirectional genetic and environmental influences on impulsivity.
